# Prognostic significance of serum MUC5AC in resected pancreatic ductal adenocarcinoma: initial insights

**DOI:** 10.3389/fonc.2025.1544928

**Published:** 2025-04-07

**Authors:** Ashish Manne, Yonghua Bao, Ankur Sheel, Amir Sara, Upender Manne, Kannan Thanikachalam, Ashwini Esnakula, Timothy M. Pawlik, Jordan M. Cloyd, Susan Tsai, Anup Kasi, Ravi Kumar Paluri, Deepak Sherpally, Sravan Jeepalyam, Lianbo Yu, Wancai Yang

**Affiliations:** ^1^ Department of Internal Medicine, Division of Medical Oncology, The Ohio State University Comprehensive Cancer Center (OSUCCC), Columbus, OH, United States; ^2^ Clinical & Translational Science Shared Resource, Comprehensive Cancer Center, The Ohio State University, Columbus, OH, United States; ^3^ Department of Pathology, University of Alabama at Birmingham, Birmingham, AL, United States; ^4^ Department of Medicine, Roswell Park Comprehensive Cancer Center, Buffalo, NY, United States; ^5^ Department of Pathology, The Ohio State University Comprehensive Cancer Center (OSUCCC), Columbus, OH, United States; ^6^ Department of Surgery, Division of Surgical Oncology, The Ohio State University Comprehensive Cancer Center (OSUCCC), Columbus, OH, United States; ^7^ Division of Medical Oncology, University of Kansas Cancer Center, Westwood, KS, United States; ^8^ Division of Hematology-Oncology, Department of Internal Medicine, Atrium Health Wake Forest Baptist Comprehensive Cancer Center, Winston-Salem, NC, United States; ^9^ Department of Internal Medicine, New York Medical College, Valhalla, NY, United States; ^10^ Department of Internal Medicine, Stormont Vail Health, Topeka, KS, United States; ^11^ Center of Biostatistics and Bioinformatics, The Ohio State University, Columbus, OH, United States

**Keywords:** pancreatic cancer, MUC5AC, biomarker, predicting, neoadjuvant therapy, FOLFIRINOX, prognostic marker, serum MUC5AC

## Abstract

**Background:**

We investigated the association between serum MUC5AC (sMUC5AC) levels and patient outcomes in individuals who underwent resection for pancreatic ductal adenocarcinoma (PDA), including those treated with neoadjuvant therapy (NAT) and those who had upfront surgery (UpS) followed by adjuvant therapy.

**Methods:**

Serum samples from the Ohio State University biorepository collected from January 2010 to June 2021 were utilized. The human MUC5AC kit (NBP2-76703) was used to perform enzyme-linked immunoassays to measure sMUC5AC levels. Logistic regression, Cox regression models (univariate and multivariate), recurrence prediction, analysis of variance (ANOVA), t-tests, and Wilcoxon tests were used for statistical analysis.

**Results:**

In the NAT cohort (n = 23), elevated sMUC5AC levels were significantly (*P* < 0.05) associated with pathological treatment response, margin positivity, and residual disease. Among 21 patients who had an R0/R1 resection (R2 resection, n=2), higher sMUC5AC levels were associated with shorter progression-free survival (PFS) (HR: 1.64, *P* = 0.0006) and overall survival (OS) (HR: 1.6, *P* = 0.005) on univariate analysis. Multivariate models confirmed sMUC5AC as an independent predictor of PFS and OS alongside pathological differentiation and postoperative therapy. Patients with lower sMUC5AC levels had more favorable pathological characteristics, better treatment responses, and improved survival outcomes. These findings were consistent in the FOLFIRINOX subgroup (n = 17). In the UpS cohort (n = 17), post-resection sMUC5AC levels tend to be associated with PFS *(P* = 0.07) and OS (*P* = 0.05). Combining sMUC5AC with Carbohydrate antigen (CA) 19-9 enhanced sensitivity (79%) and specificity (67%) to predict recurrence. Higher sMUC5AC levels were associated with earlier recurrence and poor survival outcomes, highlighting its utility in post-surgery risk stratification. Among patients with pre-treatment data (n = 11), sMUC5AC levels were significantly higher among patients with poorly differentiated tumors.

**Conclusion:**

This study provides compelling evidence for the clinical utility of sMUC5AC as a prognostic biomarker among patients with resected PDA. Future large-scale studies are needed to validate these findings and establish standard thresholds for sMUC5AC integration into clinical practice.

## Introduction

Pancreatic ductal adenocarcinoma (PDA) is associated with high mortality, even when diagnosed in the absence of metastatic disease (1). Results with the traditional treatment approach – upfront surgery (UpS) followed by adjuvant therapy (AT) to early-stage tumors [resectable (R) and borderline resectable (BR)] – have been disappointing, with high recurrence and low survival (2–11). Multiple centers now prefer neoadjuvant therapy (NAT) to treat early-stage PDA. Recently published trials (NORPACT-1, SWOG 1505, ESPAC-5, CONKO-007, A021501, PREOPANC-1/2) were largely unable to provide definitive evidence on the optimal treatment regimen, the role of radiation, and the duration of perioperative therapy (12–18). The NORPACT-1 trial noted that perioperative chemotherapy (NAT FOLFIRINOX and AT of physician’s choice) worsened overall survival (OS) versus traditional UpS/AT (23 vs. 34 months, hazard ratio (HR) of 1.46, *P*=0.158) (12). Patients treated with NAT had robust disease control (DCR, 81%) during NAT and a high objective treatment response (56%) noted in the resected sample. Patients treated with NAT also exhibited more favorable pathological features with a higher R0 resection rate (56% vs. 39%, *P*=0.018) and lower incidence of metastatic node disease (71% vs. 86%, *P*<0.001) compared with the UpS group. However, these advantages did not translate into improved OS comparing NAT versus UpS.

Patient selection beyond traditional factors are needed to inform the perioperative treatment decisions. To date, these factors have included stage of diagnosis, resectability on imaging, and serum carbohydrate antigen 19-9 (sCA19-9) levels. The poor sensitivity of sCA19-9 and the latency and evolving diagnostic uncertainty surrounding radiographic changes associated with PDA make both sCA19-9 and imaging suboptimal biomarkers to predict outcomes (19, 20). Thus, there is a need to identify novel, reliable biomarkers to help identify which patients with PDA may benefit the most from NAT and provide a real-time assessment of treatment response and aggressive tumor behavior (i.e., risk of recurrence and unfavorable pathological features). A noninvasive source for biomarkers, such as blood, may help guide treating physicians to make necessary adjustments in real-time and develop individualized strategies to improve the outcomes for patients with PDA.

Mucin 5AC (MUC5AC) is a large glycoprotein generally produced in the normal lung and gastrointestinal tracts, working alongside other mucins to shield organs from infections, inflammation, and various physiological stresses (21–25). MUC5AC is believed to play an important role in the malignant transformation of pancreatic cells (26–32). We previously studied its diagnostic value, summarized preclinical evidence suggesting its influence on treatment response, and proved its prognostic value in resected PDA post-NAT (33–37). MUC5AC detected in PDA tissue can be broadly divided into 2 major categories: mature MUC5AC (MM) detected in the apical region intracellularly and in the extracellular space (EC-M), and immature MUC5AC (IM) primarily detected in the perinuclear region (30). Our recent work demonstrated that tissue MM expression levels and site of detection in resected PDA post-NAT impact progression-free survival (PFS) (33). Expanding on these findings, we sought to further explore MUC5AC’s clinical relevance beyond tissue-based assessments by investigating the prognostic and predictive value of circulating serum MUC5AC (sMUC5AC). Prior studies have demonstrated the diagnostic value of sMUC5AC, but its prognostic (survival) and predictive (treatment response) value was never clearly defined (38–41). Preclinical and clinical studies have established MUC5AC’s role in conferring aggressive features to pancreatic cancer cells, including viability, anchorage-independent growth, motility, adhesion, angiogenesis, invasion, metastasis, and chemoresistance (35, 42–47). Given this evidence, we hypothesized that sMUC5AC could serve as a minimally invasive biomarker to improve risk stratification and treatment monitoring in PDA.

The current study aimed to further define the clinical significance of sMUC5AC in the management of resected PDA, with a specific focus on sMUC5AC levels during NAT prior to surgery. Additionally, we assessed the relevance of sMUC5AC in the postoperative period for the UpS population and at the time of diagnosis among resected patients including both patients who received NAT versus UpS.

## Materials and methods

### Study design and population

This retrospective study was conducted at The Ohio State University Comprehensive Cancer Center (OSUCCC), after receiving appropriate Institutional Review Board approvals. The Total Cancer Care Program (TCCP), a division of OSUCCC, identified the patients who underwent resection for PDA at our institution and provided serum samples from the requested study period (January 2010 to June 2021). Manual electronic medical chart review collected any additional clinical or pathologic information. For the current study, we focused on three patient populations who underwent resection of PDA: (1) the NAT group, which included patients with serum samples available for sMUC5AC testing while receiving NAT, (2) the UpS group, consisting of patients with serum samples available for sMUC5AC testing before receiving the first dose of AT, and (3) pre-treatment group with patients with serum samples available before any therapy or surgery. The key inclusion criteria for patient selection included age over 18 years, receipt of care at our institution, and consent for using their samples in research. Patients were excluded if they had a history of another or concurrent malignancy, insufficient serum for reliable sMUC5AC testing, or incomplete clinical data. None of the patients in our study had a documented history of pancreatitis or pre-malignant lesions, such as intraductal papillary mucinous neoplasms (IPMN), prior to their PDA diagnosis.

Patient-related clinical data were collected through manual chart review, and pathological characteristics were retrieved from surgical pathology reports. While categorizing pathological treatment response (pTR), patients with extensive tumors with no evident tumor regression or a response score of 3 were designated as the no-response (NR) group (48). Patients with residual tumors with evidence of regression or a response score of 2 were designated as partial response (PR) group. Patients with single cells or a small group of cancer cells or a response score of 1 were designated as near complete-response (nCR) group. The objective response (OR) group referred to patients with PR and nCR. We separately documented margin status (Ms) and residual disease status (Rd-s, categorized as R0, R1, or R2). Margin-positive (Ms-positive) indicated the presence of tumor cells at the edge of the surgical specimen. R1 disease referred to microscopically positive margins, while R2 disease indicated grossly positive margins, representing incomplete resection. In turn, Ms-positive included R1 and R2 patients. For survival analyses (PFS and OS), patients with R2 disease were excluded, as these individuals were considered advanced or palliative cases. PFS was defined as the time between the date of diagnosis and recurrence after surgery. The OS was defined as the time between the date of diagnosis and death or the last date of follow-up available at the time of data collection (July 2022). Patients with undetectable sCA19-9 levels (<15 ng/mL) on the day sMUC5AC was measured or at diagnosis were assigned the value of Zero (0).

### ELISA assay for human MUC5AC detection from human serum

Serum MUC5AC samples were analyzed using the Human MUC5AC ELISA Kit (Catalog number NBP2-76703, Novus Biologicals, Centennial, CO) following the manufacturer’s instructions. In brief, human MUC5AC protein standards in serial dilutions were prepared; 100 µL of the diluted standards or samples to MUC5AC antibody pre-coated wells of the assay plate, in triplicate, were added and incubated, followed by Biotinylated Detection anti-human MUC5AC detecting antibody, then100 μL of HRP Conjugate working solution was added, and finally, Substrate Reagents were added for incubation. The reaction was terminated by Stop Solution. Each well’s optical density (OD) value was determined at once using a microplate reader set to 450 nm. The average levels of sMUC5AC were calculated based on the serial diluted standard concentrations. The sCA19-9 level was taken from the chart review. Based on the collection dates of the samples available for sMUC5AC measurement, we identified the required sCA19-9 levels from the patient charts. The sCA19-9 value for undetectable (<15 ng/ml) patients was taken as 0 for analysis.

### Statistical considerations

Descriptive statistics were employed to summarize baseline patient characteristics, providing an overview of the study population. Univariate logistic regression models evaluated associations between pathological features and sMUC5AC levels. PFS and OS were analyzed using univariate and multivariate Cox proportional hazards regression models with clinical and pathological variables included in the multivariate adjustments to account for potential confounders. Factors significant (*P* < 0.05) on univariate analysis (UVA) were done on multivariate analysis (MVA). Analysis of variance (ANOVA), t-tests, and Wilcoxon tests were used for comparisons of the sub-groups. All statistical analyses were performed using SAS version 9.4 (SAS Institute, Cary, NC, USA).

## Results

We divided this section based on the population tested.

### Role of sMUC5AC in patients receiving NAT

The baseline pathologic features of 23 patients in this group are outlined in **Supplementary Figure S1**. The median time for serum collection was 5 weeks post-therapy (range: 4 days to 21 weeks). Serum samples were obtained from 4 patients within ≤2 weeks, 9 patients between 2–8 weeks, and 10 patients beyond 8 weeks following the initial chemotherapy dose. All patients receiving Gem-NP had their samples collected beyond eight weeks post-treatment initiation, while those treated with FOLFOX had their sample taken during week five. Among FOLFIRINOX-treated patients, one provided a sample within the first week, three had samples collected on the day of their second dose (prior to infusion), eight provided samples between weeks two and seven, and seven had samples taken after week eight. The mean sMUC5AC level was 1.82 ng/mL, with a median of 0.7 ng/mL (range: 0.4–8.3). Most patients (19/23, 83%) had sCA19-9 levels recorded on the same day as sMUC5AC measurement, while 3 patients had sCA19-9 measured 2 days later, and 1 patient had it measured 4 days later. The mean sCA19-9 level was 698 ng/mL, with a median of 181 ng/mL (range: 0–5874 ng/mL). In the FOLFIRINOX subgroup (n=19), the mean sMUC5AC level was 1.74 ng/mL, with a median of 0.7 ng/mL (range: 0.43–8.3). Patients in the NAT group received a median of 6 therapy cycles (range: 4–9), which was consistent with the FOLFIRINOX subgroup (range: 2–9 cycles).

#### sMUC5AC level during NAT is associated with clinicopathological features in the resected sample

Logistic regression analysis evaluated the association between pathological features in the resected samples and sMUC5AC levels (**Table 1**). An association was identified between sMUC5AC levels and MM expression (positive correlation), pTR, Ms, and Rd-s. There was also a trend for IM (positive correlation) and EC-M detection. In the FOLFIRINOX subgroup, pTR, Ms, and Rd-s were also associated with sMUC5AC levels, but there was no association with intracellular MUC5AC and EC-M.

**Table 1:** Univariate logistic regression of serum MUC5AC on clinicopathological features.

Pathological feature	In all NAT (N=23) *P*-value*	FOLFIRNOX (N=19) *P*-value*
Pathological differentiation, G1-2 vs G3	0.4	0.9
Peripancreatic invasion	0.6	0.2
Pathological treatment response**	**0.01**	**0.0013**
Lymphovascular invasion	0.4	0.3
Perineural invasion	0.1	0.1
**Margins-status^#^**	**0.002**	**0.007**
**Residual disease status,**	**0.002**	**0.008**
Tumor size (≤ 2 cms vs. > 2cm)	0.2	0.2
Node-status (negative vs. positive)	0.2	0.1
Premalignant lesion	0.6	0.4
Neoadjuvant CRT	0.5	0.8
**Mature MUC5AC expression (H-score)**	**0.04**	0.2
Immature MUC5AC expression **(H-score)**	0.07	0.2
**Extracellular MUC5AC detection**	**0.05**	0.1

*P-values of > 0.07 are not mentioned, # in resected sample; **nCR vs. PR vs. NR; #positive vs. negative; ^R0 vs. R1 vs. R2.

NAT, neoadjuvant therapy; G, grade (G1- well, G2-moderate, G3- poor), nCR, near complete response; PR, partial response; NR, no response; CRT, chemoradiation.

Bold, P-value is statistically significant.

To further investigate the association between sMUC5AC levels and pathological features in the resected specimens, the NAT cohort was stratified by these features, and mean sMUC5AC levels were analyzed (**Table 2**). Higher sMUC5AC levels were observed in Ms-positive patients compared with Ms-negative patients, in R2 patients compared with R0 and R1 patients, and EC-M positive versus EC-negative. In contrast, sCA19-9 levels did not differ across these groups. However, sCA19-9 levels were higher in Ms-positive patients compared with Ms-negative patients, in R1 patients compared with R0 and R2 patients, and in EC-M-positive patients versus EC-M-negative patients. sMUC5AC levels did not differ among the nCR, PR, and NR groups (p>0.05).

**Table 2:** Serum MUC5AC (mean) distribution and clinicopathological features.

	NAT-group		FOLFIRINOX-group	
	Distribution (n)	sMUC5AC levels* (*P*-value)	Distribution (n)	sMUC5AC levels* (*P*-value)
		sCA19-9 levels (*P*-value)		sCA19-9 levels (*P*-value)
Margin status (Positive vs. negative)	13 vs. 10	**2.7 vs. 0.67 (0.01)**	10 vs. 9	**2.67 vs. 0.69 (0.04)**
		810 vs. 528 (0.6)		808 vs. 570 (0.7)
Residual disease (R0 vs. R1 vs. R2)	10 vs. 11 vs. 2	**0.67 vs. 2.2 vs 5.3 (0.006)**	9 vs. 8 vs. 2	**0.69 vs. 2.02 vs. 5.35 (0.01)**
		29 vs. 952 vs. 528 (0.6)		29 vs. 1003 vs. 570 (0.6)
EC-M	14 vs. 9	2.4 vs. 0.6 (0.05)	11 vs. 8	2.3 vs. 0.9 (0.1)
Positive vs. Negative		916 vs. 332 (0.2)		1065 vs. 161 (0.1)
Treatment response (nCR vs. PR vs. NR)	2 vs. 9 vs. 12	0.43 vs. 2.84 vs. 1.28 (0.1)	2 vs. 7 vs. 10	***0.43 vs. 3.2 vs. 0.9 (0.05)***
		129 vs. 258 vs. 958 (0.4)		129 vs. 227 vs. 1061 (0.3)

EC-M, Extracellular MUC5AC; nCR, near complete response; PR, partial response; NR, no response.

Bold, P-value is statistically significant; Italics, P- value has a trend towards significance.

In the FOLFIRINOX subgroup, differences (*P*<0.05) in sMUC5AC levels were observed for Ms and Rd-s groups. No differences were noted between EC-M-positive and EC-M-negative groups in this subgroup. However, a trend toward significance was observed among nCR, PR, and NR groups, with higher sMUC5AC levels noted among PR patients. The association between pTR and other pathological features was examined to investigate potential correlations that might explain the elevated sMUC5AC levels observed in the PR group of the FOLFIRINOX cohort. While no associations were identified, a higher proportion of PR patients were Ms-positive (nCR vs. PR vs. NR = 50% vs. 71% vs. 40%, *P*= 0.4) and had peripancreatic tissue invasion (PPI) (50% vs. 71% vs. 60%, *P* = 0.8), which could potentially contribute to the increased sMUC5AC levels in this group. Finally, sMUC5AC levels did not show significant differences when stratified by other pathological features, including pathological differentiation, PPI, lymphovascular invasion (LVI), perineural invasion (PNI), tumor size (T-staging), nodal status, the presence of premalignant lesions (PML), or treatment with chemoradiation in NAT-group or FOLFIRINOX-sub group.

#### Association of sMUC5AC level with outcome in resected PDA

Among patients who underwent an R0/R1 resection (n=21), the median PFS and OS were 7.6 months and 15 months, respectively. sMUC5AC levels impacted both PFS and OS (**Table 3**). High sMUC5AC levels and poor pathological differentiation (G3) were associated with reduced survival, whereas postoperative therapy (5-FU or gem-based) improved survival outcomes. In MVA including these factors, sMUC5AC remained a predictor of PFS (**Table 4**) and a trend toward predicting OS (*P* = 0.05). Poor pathological differentiation (G3 vs. G1-2) affected PFS but not OS, while postoperative therapy significantly influenced both PFS and OS.

**Table 3:** Univariate analysis for progression-free survival in neoadjuvant therapy group (n=21).

Factor tested	Progression-free survival		Overall survival	
	*P*-value	HR (95% CI):	*P*-value	Hazard ratio (95% CI):
**Serum MUC5AC level**	**0.0006**	**1.64 (1.14 – 2.4)**	**0.005**	**1.6 (1.1 – 2.3)**
CA19-9 on the same day*	0.3		0.1	
CA19-9 at diagnosis	0.08		0.7	
**Pathological differentiation,** **G3 vs. G1-2**	**0.0007**	**4.3 (1.5 – 12.4)**	**0.02**	**3.6 (1.2 – 10.08)**
Lymph vascular invasion	0.2		0.8	
Perineural invasion	0.8		0.8	
Margins	0.1		0.3	
Tumor size, ≤ 2cms vs. 2 cms	0.5		0.8	
Node status (N0 vs. N1-N2)	0.8		0.7	
Association with premalignant lesions	0.4		0.9	
Peripancreatic invasion	0.4		0.5	
NAT CRT, Yes vs. No	0.8		0.4	
Pathological treatment response	0.8		0.7	
**Postoperative therapy received** **(5FU-based vs. Gem-based vs. none)**	***0.002***	5FU vs. Gem- 0.6, P=0.4	**0.02**	5FU vs. Gem - 0.5, P=0.3
		***5FU vs. None – 0.06, P=0.0008 (0.01 – 0.3)***		***5FU vs. None - 0.1, P=0.009 (0.01 – 0.34)***
		***Gem vs. None – 0.1, P=0.0002 (0.02 – 0.4)***		***Gem vs. None – 0.14, P=0.002 (0.03 – 0.49)***
NAT combination received^#^	0.1		0.4	

*Same day as serum MUC5AC; # FOLFIRINOX vs. FOLFOX vs. Gem/nab-paclitaxel. PFS – progression-free survival; OS – overall survival; CA19-9 – serum carbohydrate antigen 19-9, G- grade (G1- well, G2-moderate, G3- poor); NAT, neoadjuvant therapy; CRT, chemoradiation; 5FU, 5-fluorouracil; Gem, gemcitabine.

Bold, P-value is statistically significant; Italics, P- value has a trend towards significance.

**Table 4:** Multivariate analysis for neoadjuvant therapy cohort.

Source	*P*-value	Level1	/Level2	Hazard Ratio	p-value	Lower	Upper
PFS							
Serum MUC5AC level	**0.0467**			1.478755		2.21634	0.6762443
Path diff	**0.0480**	G3	G1-2	3.2366587		1.0102253	10.369924
Postoperative therapy	**0.0185**	5FU-based	Gem-based	0.8329576	0.7607	0.2569264	2.7004563
		5FU-based	None	**0.1068042**	**0.0076**	0.020651	0.5523779
		Gem-based	None	**0.1282228**	**0.0097**	0.0270236	0.6083967
Overall survival							
Serum MUC5AC level	***0.0539***			1.433842		0.994179	2.121352
Path diff G1-2 vs G3	0.3284			1.8524537		0.5380476	6.3778455
Postoperative therapy	**0.0125**	5FU-based	Gem-based	0.6757719	0.5321	0.1976299	2.3107211
		5FU-based	None	**0.1000313**	**0.0050**	0.0200773	0.4983876
		Gem-based	None	**0.1480252**	**0.0095**	0.034921	0.6274577

Path diff, pathological differentiation; G, grade (G1- well, G2-moderate, G3- poor); 5FU, 5-fluorouracil; Gem, gemcitabine.

Bold, P-value is statistically significant.

A similar analysis in the FOLFIRINOX subgroup (n=17) yielded comparable results in univariate analysis (UVA, **Supplementary Table S4**). In this group, postoperative therapy significantly impacted both PFS and OS in MVA (**Supplementary Table S4**). sMUC5AC influenced OS but did not have a significant effect on PFS. Poor pathological differentiation showed a trend toward affecting PFS but was not associated with OS.

#### Pre-surgery models

We evaluated various models (**Table 5**) integrating sMUC5AC with the pathological differentiation (G1-2 vs. G3) as these are the two clinical factors accessible to the physician administering NAT. Our objective was to develop models to predict recurrence risk before surgery. sMUC5AC impacted PFS and OS significantly. Pathological differentiation played a role in PFS but had no impact on OS. Building on the established clinical value of combining sMUC5AC and sCA19-9 in diagnosis, we evaluated additional MVA models, including it (on the day of sMUC5AC measurement and at diagnosis) (38, 41). The results reaffirmed the prognostic value of sMUC5AC (**Supplementary Tables S4, S5**), which demonstrated a negative impact on survival, with increased risk of death (HRs ranging from 1.4 to 1.7).

**Table 5:** Presurgery Multivariate analysis for survival.

Source	*P*-value	Hazard Ratio	Lower 95%	Upper 95%
Progression-free Survival				
Serum MUC5AC level	**0.0269**	1.51557	1.041124	2.231702
Path diff, G3 vs G1-2	**0.0283**	3.553659	1.144105	11.03789
Overall Survival				
Serum MUC5AC level	**0.0459**	1.440818	1.002623	2.105105
Path diff, G3 vs G1-2	0.1116	2.617977	0.800053	8.566686

Path diff, pathological differentiation; G, grade (G1- well, G2-moderate, G3- poor).

Bold, P-value is statistically significant.

#### High vs. low MUC5AC groups

**Figure 1:**
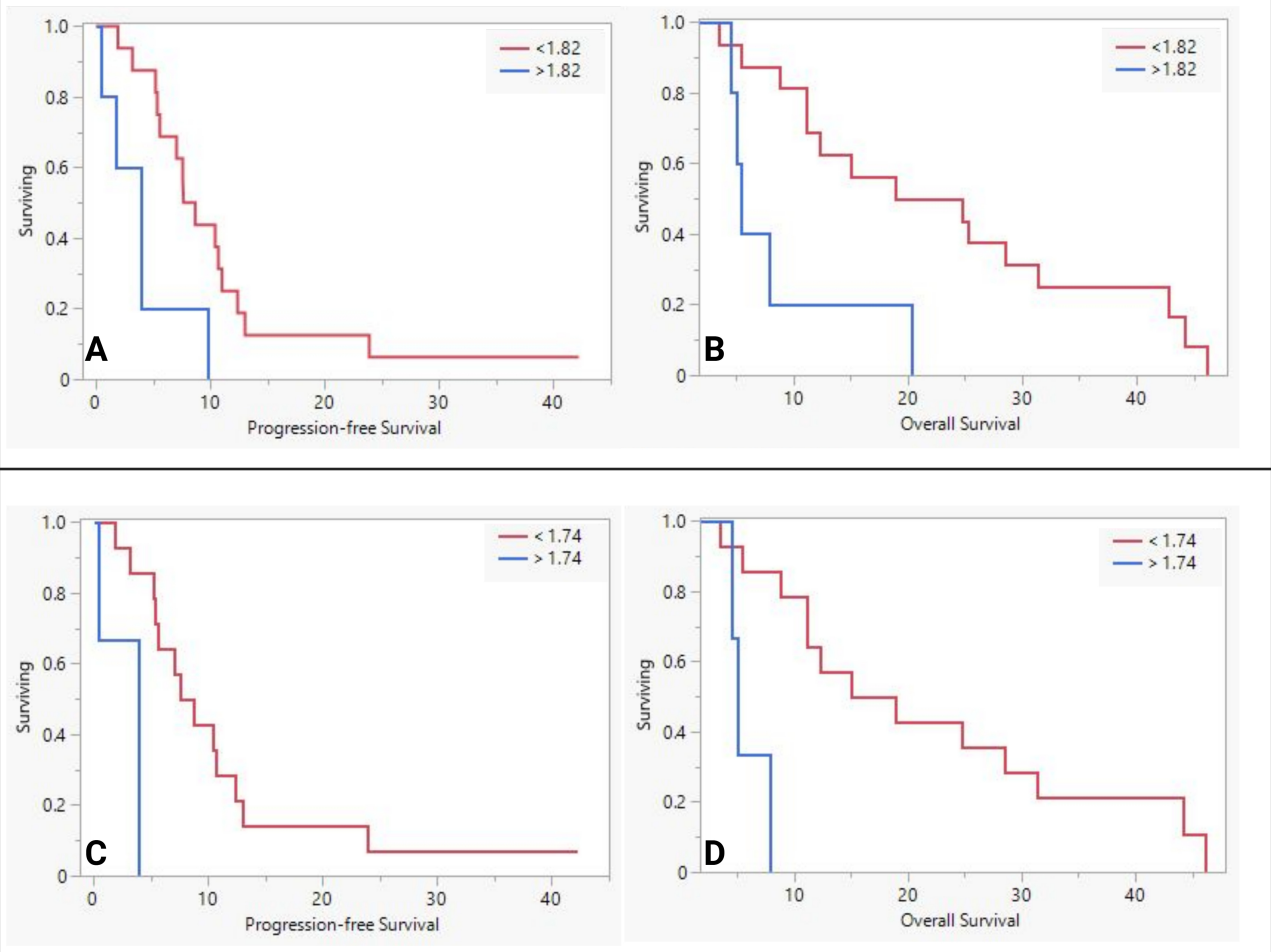
The difference in survival between low and high serum MUC5AC groups in neoadjuvant therapy group **(A, B)** and FOLFIRINOX sub-group **(C, D)**.

We further examined the impact of MUC5AC by dividing the NAT based on the means in their respective groups as thresholds (**Table 6**). The lower sMUC5AC group (n=16) had higher fraction of patients with larger (>2 cm) tumors, R0, and Ms-negative disease than the higher sMUC5AC group (n=7). The lower group also had better survival (PFS and OS, see **Figures 1A, B**). Similar results (**Table 6**, **Figures 1C, D**) were observed in the FOLFIRINOX subgroup (n=19, the mean sMUC5AC (in ng/mL) was 1.74, with a median of 0.7 (range of 0.43-8.3)).

**Table 6:** Comparing low and high MUC5AC groups.

Factors tested	NAT-group (*P*-value)*	FOLFIRINOX (*P*-value)*
**Threshold MUC5AC in ng/mL (N)**	≤ 1.82 (n=16) vs. >1.82 (n=7)	≤ 1.74 (n=14) vs. > 1.74 (n=5)
CA19-9 mean (ng/mL)	831 vs. 361	817 vs. 358
nCR vs. PR vs. NR (%)^L^	***100 vs. 44 vs. 83 (0.07)***	***100 vs. 43 vs. 90 (0.05)***
OR (%)	38 vs. 71 (0.1)	*36 vs. 80 (0.08)*
Mature MUC5AC expression (H-score)	118 vs. 180 (0.1)	131 vs. 162
Immature MUC5AC expression (H-score)	119 vs. 163 (0.1)	133 vs. 150
EC-mature MUC5AC-detection %	50 vs. 86 (0.1)	50 vs. 80
EC-M CS	85 vs. 176 (0.08)	96 vs. 156
Pathological differentiation^#^	69% vs. 71%	70 vs. 67
**Tumor size, ≤ 2 cm vs. > 2 cm,** **% of patients with > 2 cm**	**88 vs. 43 (0.02)**	**86 vs. 40 (0.04)**
**Residual disease, R0 vs. R1 vs. R2^L^**	**100 vs. 55 vs. 0 (0.001)**	**100 vs. 62.5 vs. 0 vs. (0.003)**
**Margin-positive %**	**38 vs. 100 (0.001)**	**50 vs. 100 (0.004)**
Node positive %	82 vs. 57	79 vs. 40 (0.1)
Perineural invasion-positive %	94 vs. 71	*93 vs. 60 (0.08)*
Peripancreatic extension	56 vs. 57	65 vs. 60
Lymph vascular invasion- positive %	63 vs. 49	57 vs. 20 (0.1)
**Progression-free survival^ (in months)**	**8 vs. 4 (0.006)**	**8 vs. 4 (0.003)**
**Overall survival^ (in months)**	**22 vs. 5.3 (0.008)**	**17 vs. 5 (0.003)**

*If the *P*-value is >0.1, it is not reported; ^#^G1-2 vs. G3; L- in lower compared to higher; ^removed patients with R2 disease.

MUC5AC, serum MUC5AC level; CA19-9, serum carbohydrate antigen 19-9; nCR, near complete response; PR, partial response; NR, no response; OR, objective response; EC-M CS, extracellular MUC5AC composite score.

Bold, P-value is statistically significant; Italics, P- value has a trend towards significance.

### sMUC5AC as a predictor for recurrence post-surgery

We evaluated 17 patients with serum samples available post-surgery (UpS group) and before the first dose of AT. We discussed baseline pathological features in **Supplementary Table S1**. Most (17/19) had sCA19-9 levels from the same day as sMUC5AC levels. One had sCA19-9 1 day before, and another had it 1 day after the sMUC5AC collection date. The median time interval between serum collection and surgery was 8 weeks (range: 2–12 weeks), while the interval between serum collection and chemotherapy was 2 weeks (range: <1–12 weeks). The mean sMUC5AC level was 1.16 (median 0.87 ng/mL, range of 0.42-3.3). The mean sCA19-9 level was 492 (median 29.52 ng/mL, range of 0-4785).

The PFS and OS of the cohort were 15m and 26m, respectively. sMUC5AC demonstrated a trend toward significance for PFS (*P*=0.07) and OS (*P*=0.05) on UVA (**Supplementary Table S6**). sCA19-9 levels on the same day, pathological differentiation, LVI, PPI, and EC-M expression significantly impacted PFS in UVA. Similarly, LVI and PPI were associated with OS on UVA. The effect (positive vs. negative) of these factors was on the expected lines except for EC-M. It had a positive impact on PFS, which is opposite to the effect it had on the NAT population in our previous study (33).

sMUC5AC maintained its significance in the tested MVA models for PFS and OS (see **Table 7**). LVI for PFS and PPI for OS had a trend toward significance. EC-M did not impact the PFS. We examined several post-surgery MVA models, drawing insights from the pre-surgery modeling exercise (**Supplementary Table S7**). SMUC5AC was significant for both PFS and OS in those models too.

**Table 7:** Multivariate analysis of upfront surgery population for survival (n=17).

Factor tested	*P*-value	Hazard Ratio	Lower 95%	Upper 95%
Progression-free survival				
**Serum MUC5AC level**	**0.0268**	**3.148134**	**1.170522**	**9.922203**
Serum CA 19-9 on the same day	0.6063	1.00037	0.998841	1.001743
Path diff G1-2 vs G3	0.5774	2.592731	0.5774	0.090809
***LVI, yes vs. no***	***0.0753***	***4.734761***	***0.0753***	***0.853188***
PPI, yes vs. no	0.954	1.101294	0.954	0.04148
Extracellular MUC5AC, Positive vs. negative	0.358	0.19543	0.358	0.006014
Overall survival				
**Serum MUC5AC level**	**0.0374**	**2.217179**	**1.012598**	**4.877902**
LVI, yes vs. no	0.2258	4.128347	0.416315	40.93837
***PPI, yes vs. no***	***0.0542***	***4.225132***	***0.974331***	***18.32205***

CA19-9, serum carbohydrate antigen 19-9; Path diff, pathological differentiation; G, grade (G1- well, G2-moderate, G3- poor); LVI, lymphovascular invasion; PPI, peripancreatic invasion.

Bold, P-value is statistically significant; Italics, P- value has a trend towards significance.

**Figure 2:**
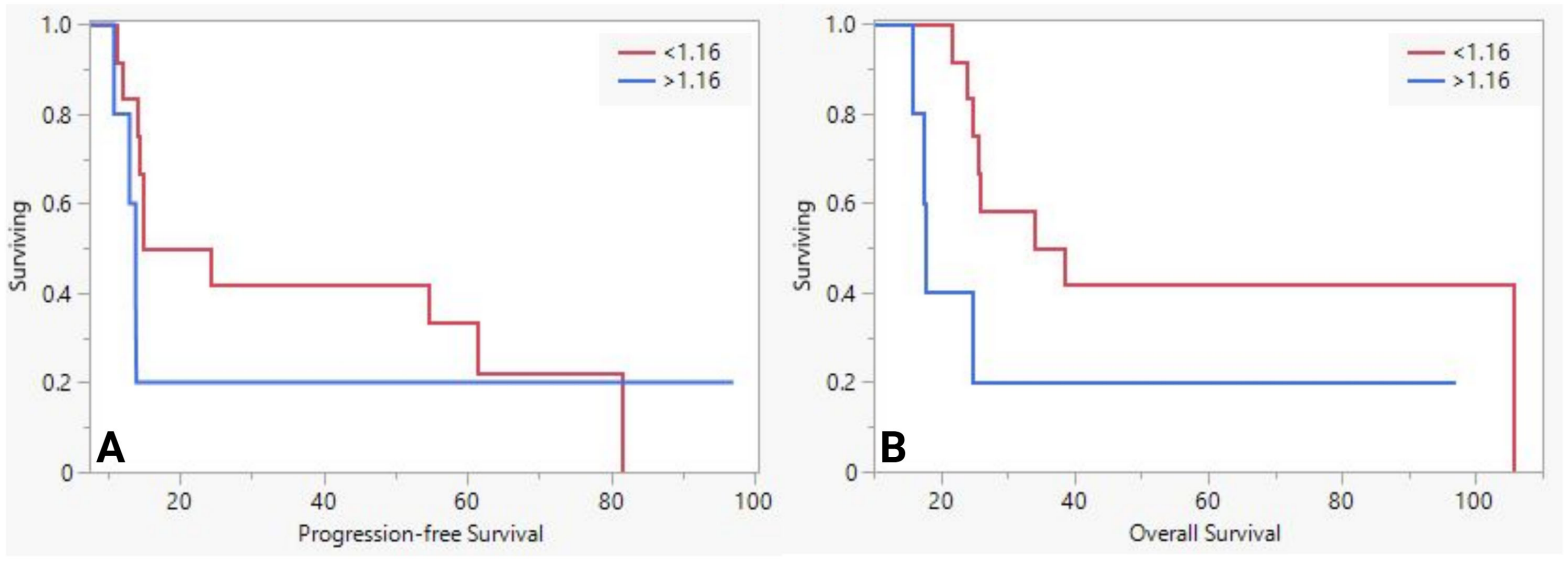
The difference in progression-free survival **(A)** and overall survival **(B)** between low and high serum MUC5AC groups in upfront surgery group.

We stratified the population based on the mean sMUC5AC levels and analyzed them as we did for NAT-group. While PFS was similar between the groups (20m vs. 14m, *P*=0.1), OS was significantly worse (**Figures 2A, B** in the group with higher sMUC5AC levels (36 vs. 18. *P*=0.02). No significant differences in pathological features were observed between the two groups.

Next, we evaluated the ability of sMUC5AC to predict recurrence (**Table 8**). Recurrence prediction was better when sMUC5AC (mean or median of this study) was combined with sCA19-9 (abnormal > 37 ng/mL).

**Table 8:** Predicting recurrence using MUC5AC and CA19-9 post-surgery.

	Mean* sMUC5AC	Median^#^ sMUC5AC	Abnormal sCA19-9^	Mean sMUC5AC + sCA19-9	Median sMUC5AC+ sCA19-9
Sensitivity (%)	29	50	36	58	79
Specificity (%)	67	67	100	100	67
PPV (%)	80	88	100	100	92
NPV (%)	17	22	25	33	40
Accuracy (%)	35	53	47	65	76

*Mean of 1.16ng/mL as cut-off; ^#^median of 0.87 ng/mL as cut-off; ^^^CA19-9 >37 U/mL.

PPV, positive predictive value; NPV, negative predictive value; sCA19-9, serum carbohydrate antigen 19-9; sMUC5AC, serum MUC5AC.

Finally, no significant associations were identified between the pathological features in the resected specimens and sMUC5AC levels, nor were there significant differences in sMUC5AC levels when stratified by pathological features in this population. These findings were in contrast with the findings observed in the NAT group (**Tables 1**, **2**).

### sMUC5AC at the diagnosis

We analyzed data from 11 patients (9 from the NAT group and 2 from the UpS group). The mean sMUC5AC level was 3.6 ng/mL, with a median of 0.8 ng/mL (range: 0.41 to 26.2 ng/mL). In the NAT group, 7 out of 9 patients received FOLFIRINOX, one received FOLFOX, and one received Gem-NP. A statistically significant positive correlation was observed between the two biomarkers (p = 0.0078; **Supplementary Figure S1**). sMUC5AC levels were significantly (*P*<0.05) associated with pathological differentiation and association with premalignant lesions. Within the cohort, comparisons demonstrated higher sMUC5AC levels in poorly differentiated tumors (G3: 13.5 ng/mL vs. G1-2: 1.3 ng/mL; n = 2 vs. n = 9) and in patients without PML (no premalignant lesions: 7.0 ng/mL vs. PanIN: 0.62 ng/mL; n = 5 vs. n = 6). We did not have enough patients in NAT or UpS groups for a meaningful survival analysis.

## Discussion

The clinical significance of tissue MUC5AC in PDA remains ambiguous, and the utility of sMUC5AC as a prognostic or predictive marker has yet to be extensively studied. Earlier studies by Kaur et al. (2016) and Zhang et al. (2020) focused on the diagnostic value of sMUC5AC, demonstrating that elevated levels in early-stage PDA compared with chronic pancreatitis and benign conditions (38, 41). The diagnostic accuracy improved when sMUC5AC was combined with sCA19-9. The current study was unique in that we examined the clinical utility of sMUC5AC to guide the management of PDA patients, particularly individuals undergoing NAT and UpS.

In the NAT population, elevated sMUC5AC during NAT (median time of 5 weeks, which could be 2-3 doses of chemotherapy) was associated with shorter PFS and OS. On MVA, sMUC5AC remained a predictor of PFS and demonstrated a trend toward worse OS. Low sMUC5AC levels were associated with better survival outcomes, improved treatment responses, and favorable pathological characteristics. Notably, sMUC5AC outperformed CA19-9, which had limited prognostic value with clinically insignificant HRs in most analyses. sMUC5AC levels correlated with key pathological features, such as pTR, Ms, and Rd. sMUC5AC levels were significantly higher in patients with Ms-positive disease and R2 resections, highlighting its potential as a biomarker for aggressive pathological features. This aligns with the preclinical evidence extensively discussed in our review (35). Notably, one study demonstrated that knocking out the MUC5AC gene in pancreatic cancer cell lines before transplantation into nude mice resulted in significantly reduced tumor weight and fewer metastatic sites, reinforcing its role in tumor progression and metastatic potential (42). In the FOLFIRINOX subgroup, elevated sMUC5AC levels significantly impacted OS and PFS on univariate analysis but OS only on MVA, consistent with findings in the broader study cohort.

Among patients who underwent UpS, post-surgical sMUC5AC testing, alone or combined with sCA19-9, can guide surveillance and management. sMUC5AC demonstrated a trend toward significance for predicting both PFs and OS, underscoring its value as a prognostic biomarker. While CA19-9 levels on the same day were associated with PFS, these values did not demonstrate predictive value for OS, limiting their utility for long-term outcome prediction. On MVA, sMUC5AC stood out as a significant biomarker to predict PFS and OS, outperforming CA19-9, which exhibited clinically insignificant HRs at diagnosis and on the same day of testing. While the median sMUC5AC cutoff offered moderate sensitivity and specificity, the mean cutoff achieved perfect specificity but lower sensitivity. Combining sMUC5AC with CA19-9 improved sensitivity significantly, allowing better identification of patients at risk of recurrence, though at the cost of reduced specificity.

We needed more patients with sMUC5AC measurements at diagnosis (before NAT or UpS) to evaluate its impact on outcomes thoroughly. However, the available data provided valuable insights into sMUC5AC levels in early-stage tumors at diagnosis. The median sMUC5AC (0.8 ng/mL) of the pre-treatment group was similar to NAT (0.7 ng/mL) and UpS groups (0.8 ng/mL). Still, elevated sMUC5AC levels post-NAT and post-surgery were associated with worse outcomes and adverse pathological resected samples (e.g., poor differentiation, Ms-positivity, Rd, LVI, and PPI). Based on our observations from these preliminary data, we postulate that MUC5AC plays a central role in driving the aggressiveness of pancreatic tumor cells, with circulating sMUC5AC levels reflecting its activity at a given time point.

As NAT becomes increasingly adopted as the initial approach for early-stage and certain locally advanced pancreatic tumors, data from the current study provide the first compelling evidence supporting perioperative sMUC5AC as a valuable prognostic biomarker in this population (49).

sMUC5AC has the potential to complement existing tools, such as sCA19-9 and imaging, which have suboptimal sensitivity and specificity. Incorporating sMUC5AC testing into clinical practice could improve post-operative therapy decisions, particularly for patients undergoing extended NAT (>4 months). Currently, no studies in the literature have examined the correlation between sCA19-9 and sMUC5AC levels in patients receiving systemic therapy. However, in our cohort, some patients (3 in NAT group and 9 in UpS group) with undetectable sCA19-9 had measurable sMUC5AC levels, highlighting its potential as a complementary biomarker. This is particularly relevant in the context of NAT, where fluctuations in sCA19-9 may not reliably reflect tumor burden or treatment response. By offering an additional biomarker for disease monitoring, sMUC5AC could help refine real-time treatment decisions, potentially guiding the intensification or modification of therapeutic strategies based on response. Furthermore, in the post-treatment surveillance setting, sMUC5AC may serve as a valuable adjunct to sCA19-9, particularly in patients with CA19-9 non-secreting tumors, where conventional biomarkers may not be informative. With the increasing interest in circulating tumor DNA (ctDNA) for recurrence and resistance monitoring, integrating sMUC5AC with ctDNA assays could enhance the predictive accuracy of biomarker-driven surveillance strategies. A multimodal approach incorporating sMUC5AC, sCA19-9, and ctDNA could lead to more robust risk stratification, enabling earlier detection of recurrence and more personalized treatment planning.

Our findings in the UpS group should be further evaluated in more extensive studies to validate their true clinical significance. If confirmed, sMUC5AC could serve as a valuable biomarker for detecting early disease recurrence or treatment resistance in this population, enabling timely therapeutic adjustments during adjuvant chemotherapy with regimens such as FOLFIRINOX or Gem/Cap (2–11). These findings also have implications for advanced-stage tumors, in which sMUC5AC could be a tool to monitor treatment resistance. The results also provide preliminary evidence supporting MUC5AC as a potential therapeutic target. Future strategies aimed at modulating MUC5AC expression may help improve PDA outcomes.

This study has limitations that should be acknowledged. First, the small sample size of our single-center retrospective study restricts the generalizability of the findings and may have limited power to detect differences in the study groups. Second, the timing of sample collection for sMUC5AC testing was inconsistent among study participants, potentially impacting the reliability of the biomarker analysis. Additionally, variability in postoperative therapies among patients could have influenced outcomes, making it difficult to isolate the impact of sMUC5AC on survival and recurrence. Measurement challenges were another limitation. For instance, sCA19-9 values below 15 ng/mL were recorded as 0 ng/mL, which may have reduced the accuracy of the data for low-level readings. A clear and objective distinction between BR and R PDA was not established in the study population, limiting our ability to analyze the association of sMUC5AC with tumor staging. As described in the Material and Methods, to standardize the ELISA results, standard serum aliquots were freshly thawed and serial dilutions (1:3 dilution and 6 different dilutions) were prepared starting with the highest dilution as the manufacturer recommended. These were applied to the wells on the ELISA plate, and 3 blank wells (assigned with diluent buffer) were also designated. All tested values were calculated based on the standard curve and OD reads by reduction of blank/background. Moreover, the ELISA method used in this study is less sensitive than multiplex assays, potentially limiting the detection of subtle fluctuations in sMUC5AC levels. Furthermore, serial samples were unavailable, preventing us from analyzing dynamic changes in sMUC5AC levels from diagnosis through NAT and post-surgery. This limitation hindered our ability to fully explore the dynamic relationship between sMUC5AC levels, treatment response, and long-term outcomes. Despite these challenges, this study provides valuable preliminary data highlighting the potential of sMUC5AC as a prognostic biomarker in PDA. Future research with larger, multicenter cohorts, standardized sample collection protocols, and advanced assays will be crucial to validate these findings. Continued exploration of sMUC5AC in conjunction with pathological and clinical factors holds significant promise for improving predictive models for recurrence and survival in PDA management.

## Conclusion

Data from the current study highlights the potential significance of sMUC5AC levels in patients with resected PDA. sMUC5AC improves the accuracy of predictive models for recurrence and survival combined with CA19-9 and pathological factors (enhanced risk stratification). Elevated sMUC5AC levels can help identify patients who may benefit from more aggressive post-operative therapies or wider resection margins during surgery (treatment guidance). Regular sMUC5AC testing post-surgery could provide a reliable tool for recurrence monitoring, complementing or replacing standard imaging and sCA19-9 (post-surgical surveillance). However, larger multicenter prospective validation studies are essential to confirm these findings across diverse patient populations and clinical settings to establish its clinical utility fully. Additionally, integrating sMUC5AC testing into existing clinical workflows will require further investigation to optimize its implementation and ensure seamless adoption in routine oncologic care.

## Data Availability

The private dataset used in this study is not readily available due to restrictions imposed by ethical guidelines and data privacy regulations. Requests to access the dataset should be directed to AM at ashish.manne@osumc.edu
