# Supplementary material for: Prognostic significance of serum MUC5AC in resected pancreatic ductal adenocarcinoma: initial insights
Source: Front Oncol. 2025 Apr 7;15:1544928. doi: 10.3389/fonc.2025.1544928 (PMC12010103; doi:10.3389/fonc.2025.1544928)
Supplement: Supplementary file 1 [file DataSheet1.pdf]

Supplementary Material

Supplementary Table 1: Baseline characteristics of the population studied

Pathological feature	Distribution in percentages (%)	
	NAT-group (n=23)	UpS (n=17)
Median age	66 years; (range 43- 81)	66 years (range 47- 84)
Gender	Male – 48<br>Female – 52	Male – 42<br>Female – 68
Race	Caucasian – 92<br>African-American – 4<br>Asian - 4	Caucasian – 95<br>African-American – 5
Differentiation (G1 vs. G2 vs. G3)	4 vs. 65 vs. 31	17 vs. 59 vs. 24
Peripancreatic extension	57	35
Treatment effect noted nCR vs. PR vs. NR	9 vs. 39 vs. 52	Not applicable
Tumor size, ≤ 2 cm vs. 2-4 cm vs. > 4 cm	31 vs. 43 vs. 26	35 vs. 47 vs. 18
Lymph vascular invasion identified	57	71
Perineural invasion identified	87	82
Margins positive (n=10)	57	12
Residual disease: R0 vs. R1 vs. R2	43 vs. 48 vs. 9	88 vs. 12 vs. 0
Node positivity,	74	76
Association with premalignant lesion:<br>PanIN vs. IPMN vs. no lesion identified	8vs. 22 vs. 70	11 vs. 68 vs. 21
Neoadjuvant chemoradiation	35	Not applicable
Adjuvant chemotherapy[R2] (n=21)	Gem – 14<br>Gem/Cap – 14<br>Gem/NP – 10<br>5FU – 5<br>FOLFIRINOX – 24<br>None – 33	Gem – 41<br>Gem/Cap – 24<br>Gem/NP – 24<br>Cap – 6<br>CRT – 5

R2 – one patient with R2 received Gem/NP, and the other died before receiving systemic therapy in the NAT-group. They are not considered to have adjuvant therapy.

NAT – neoadjuvant therapy, UpS – upfront surgery, nCR- near complete response, OR - Objective response, NR - no response, cm – centimeters, PanIN—pancreatic intraepithelial neoplasia; IPMN—intraductal papillary mucinous neoplasms. Gem – gemcitabine, cap – capecitabine, NP – nab-paclitaxel, FU – furouracil.

Supplementary Table 2: Univariate analysis for overall survival in the FOLFIRINOX group (n=17)

	FOLFIRINOX group			
**Factor tested**	**PFS**	**HR**	**OS**	**HR**
**Serum MUC5AC level**	**0.03**	**1.5**	**0.01**	**1.6**
CA19-9 on the same day*				
CA19-9 at diagnosis				
**Pathological differentiation, G3 vs. G1-2**	**0.02**	**3.8**	**0.04**	**3.1**
Lymph vascular invasion				
Perineural invasion				
Margins				
Tumor size, ≤ 2cms vs. 2 cms				
Node status (N0 vs. N1-N2)				
Association with premalignant lesions				
Peripancreatic invasion				
NAT CRT, Yes vs. No				
Pathological treatment response				
**Postoperative therapy received (5FU-based vs. Gem-based vs. none)**	**0.001**	***5FU vs. Gem – NS<br>5FU vs. None - 0.1, p=0.004<br>Gem vs. None – 0.14, p=0.01***	**0.001**	***5FU vs. Gem – NS<br>5FU vs. None - 0.1, p=0.003<br>Gem vs. None – 0.14, p=0.02***
NAT combination received[#]	0.			

* Same day as serum MUC5AC; # FOLFIRINOX vs. FOLFOX vs. Gem/nab-paclitaxel.
PFS – progression-free survival, OS – overall survival, CA19-9 – serum carbohydrate antigen 19-9, G – grade (G1- well, G2-moderate, G3- poor) ; NAT – neoadjuvant therapy; CRT – chemoradiation; 5FU – 5-fluorouracil; Gem – gemcitabine, NS – not significant

Supplementary Table 3: Multivariate analysis in the FOLFIRINOX group (n=17)

Source	p-value	Level1	/Level2	Hazard Ratio	p-value	Lower	Upper
Progression-free survival							
Serum MUC5AC level	0.0947			1.46424		0.901983	2.314812
Path diff G1-2 vs G3	0.0597	G3	G1-2	3.4738938	0.0597	0.9505496	12.695747
Postoperative therapy	0.0380	5FU-based	Gem-based	1.0013947	0.9983	0.2686113	3.733244
		5FU-based	None	0.143615	0.0176	0.028917	0.7132575
		Gem-based	None	0.143415	0.0212	0.0274811	0.7484366
Overall Survival							
Serum MUC5AC level	0.0108			1.90169		1.174668	3.143655
Path diff G1-2 vs G3	0.2278	G3	G1-2	2.1984257	0.2266	0.6131042	7.8829591
Postoperative therapy	0.0051	5FU-based	Gem-based	0.562536	0.4308	0.134461	2.3534472
		5FU-based	None	0.0755421	0.0021	0.0145371	0.3925543
		Gem-based	None	0.134289	0.0152	0.026535	0.679615

Path diff- pathological differentiation, G – grade (G1- well, G2-moderate, G3- poor), 5FU – 5-fluorouracil; Gem – gemcitabine,

Supplementary Table 4: Pre-surgery Multivariate models for progression-free survival

	NAT		FOLFIRINOX	
**Factor**	**p-value**	**Hazard Ratio**	**p-value**	**Hazard Ratio**
Post-surgery model 1				
sMUC5AC	**0.01**	**1.6**	***0.05***	***1.5***
Path diff, G3 vs. G1-2	**0.02**	**4**	**0.04**	**3.85**
CA19-9 on the same day	***0.05***	***1.0003***	0.1	1.0002
Post-surgery model 2				
**sMUC5AC**	**0.003**	**1.74**	**0.02**	**1.6**
CA19-9 on the same day*	0.09	1.00025	0.1	1.0002
Post-surgery model 3				
**sMUC5AC**	**0.009**	**1.7**	**0.03**	**1.6**
Path diff, G3 vs. G1-2	***0.05***	***3.2***	0.08	3.07
CA19-9 at diagnosis	0.03	1.0002	0.05	1.0001
Post-surgery model 4				
**sMUC5AC**	**0.01**	**1.75**	**0.03**	**1.64**
Path diff, G3 vs. G1-2	**0.04**	**3.5**	0.08	3.2
CA19-9 same day	0.5	1.00001	0.7	1.00008
CA19-9 at diagnosis	0.2	1.00001	0.2	1.00001
Post-surgery model 5				
**sMUC5AC**	**0.002**	**1.9**	**0.01**	**1.76**
CA19-9 same day	0.9	1.00002	0.8	0.9
CA19-9 at diagnosis	***0.06***	***1.00002***	0.08	1.0002

* same day as serum MUC5AC

; sMUC5AC – serum MUC5AC level; CA19-9 – serum carbohydrate antigen 19-9; Path diff – pathological differentiation, G- grade (G1- well, G2- moderate, G3- poor),

Supplementary Table 5: Pre-surgery models for Overall Survival

	NAT - group		FOLFIRINOX- group	
**Factor**	**p-value**	**Hazard Ratio**	**p-value**	**Hazard Ratio**
Post-surgery model 1				
sMUC5AC	***0.06***	***1.4***	***0.05***	***1.5***
Path diff, G3 vs. G1-2	0.1	2.6	**0.04**	**3.85**
CA19-9 on the same day	*0.2*	*0.9*	0.1	1.0002
Post-surgery model 2				
**sMUC5AC**	**0.008**	**1.57**	**0.02**	**1.55**
CA19-9 on the same day*	0.3	0.9	0.32	0.9
Post-surgery model 3				
**sMUC5AC**	***0.05***	***1.4***	**0.03**	**1.6**
Path diff, G3 vs. G1-2	*0.1*	*2.7*	0.08	3.07
CA19-9 at diagnosis	0.7	0.9	***0.05***	***1.0001***
Post-surgery model 4				
**sMUC5AC**	**0.04**	**1.4**	***0.0737***	***1.47***
Path diff, G3 vs. G1-2	0.1	2.3	0.2758	2
CA19-9 same day	0.2	0.9	0.2489	0.9
CA19-9 at diagnosis	0.3	1.00134	0.3115	1.0001
Post-surgery model 5				
**sMUC5AC**	***0.007***	***1.6***	***0.01***	***1.6***
CA19-9 same day	0.1	0.9	0.1	0.9
CA19-9 at diagnosis	0.1	1.0001	0.1	1.0001

* same day as serum MUC5AC
NAT – neoadjuvant therapy,sMUC5AC – serum MUC5AC level; CA19-9 – serum carbohydrate antigen 19-9; G- grade (G1- well, G2-moderate, G3- poor).

Supplementary Table 6: Univariate analysis for survival in post-surgery patients – new (n=17)

	Progression-free survival		Overall survival	
Factor tested	p-value	Hazard ratio	p-value	Hazard ratio
Serum MUC5AC level	***0.07***	***1.93***	***0.05***	***1.9***
CA 19-9 on the same day	**0.04**	1.0007	0.8	
CA 19-9 on the diagnosis	0.5		0.08	1.0004
Pathological differentiation, G3 vs. G1-2	**0.02**	**4.3**	0.1	
Lymph vascular invasion, Yes vs. No	**0.03**	**5.4**	**0.04**	**8.3**
Perineural invasion	0.9	**2.6**	0.9	
Margins	0.7		0.4	
Tumor size, ≤2 cm vs. 2 cm	0.3		0.4	
Node status (N0 vs. N1-N2)	0.08		0.1	
Association with premalignant lesions	0.5		0.7	
Peripancreatic invasion, Yes vs. No	**0.02**	**4.5**	**0.008**	**5.3**
Adjuvant therapy (5FU-based vs. Gem-based vs. none)	0.9		0.7	
**Extracellular MUC5AC positive vs. negative**	**0.01**	**0.1**	0.8	

MUC5AC – mucin 5 AC, Ca19-9 - Carbohydrate antigen 19-9; 5FU - 5-fluorouracil, Gem – Gemcitabine; G- grade (G1- well, G2-moderate, G3- poor),

Supplementary Table 7: Post-surgery models for predicting overall survival

	**Progression-free survival**		Overall survival	
	***P*-value**	**HR**	***P*-value**	**HR**
Model 1				
**Serum MUC5AC level**	**0.02**	**2.56**	**0.04**	**1.96**
**CA19-9 on the same day**	**0.01**	**1.0001**	0.6	1.001
Model 2				
**Serum MUC5AC level**	**0.01**	**2.98**	**0.01**	**2.46**
**CA19-9 on the same day**	**0.01**	**1.0011**	0.5	1.0001
**CA19-9 at diagnosis**	**0.1**	1.0003	**0.01**	**1.0006**

MUC5AC – mucin 5 AC, Ca19-9 - Carbohydrate antigen 19-9;

Supplementary Figure 1: Correlation between pre-treatment serum MUC5AC and CA19-9

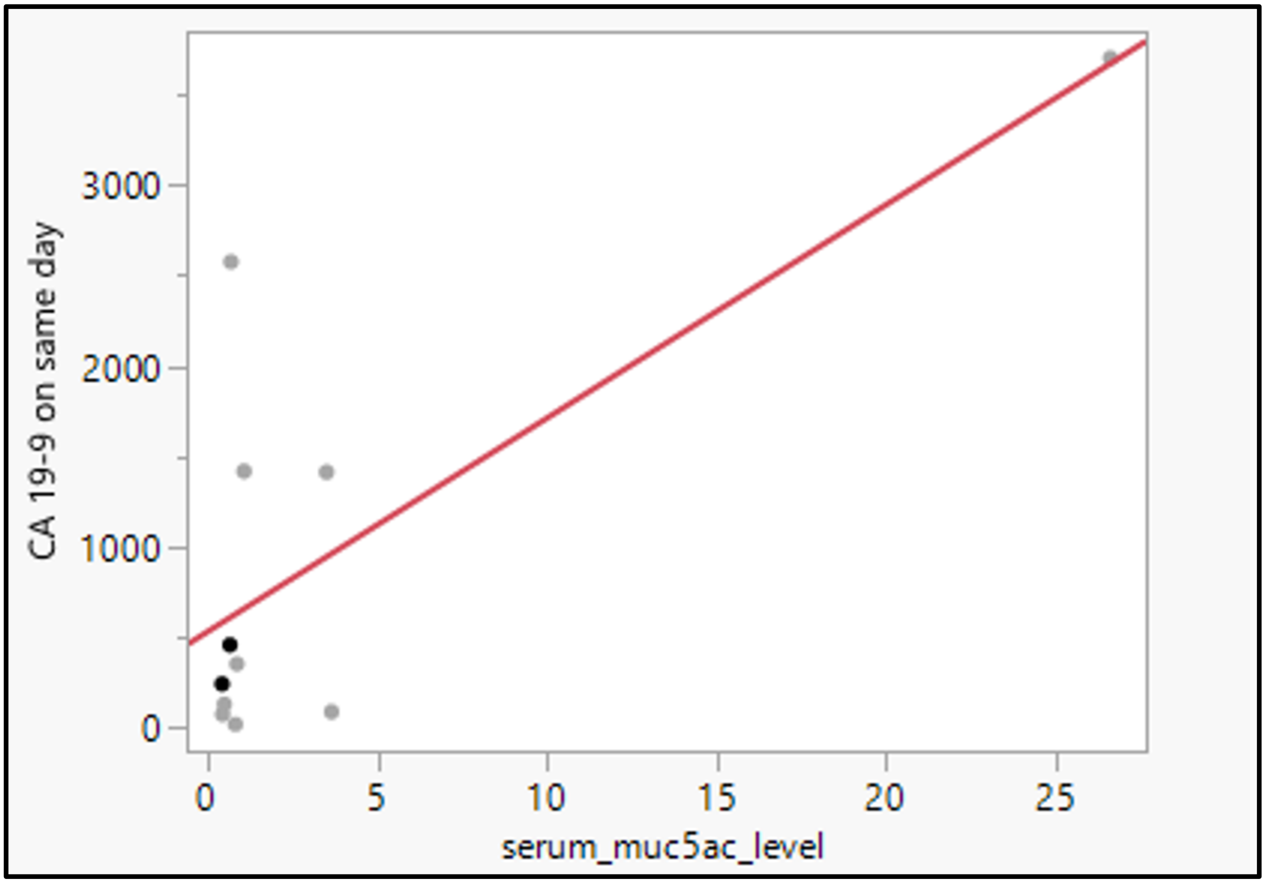

MUC5AC – mucin 5 AC, Ca19-9 - Carbohydrate antigen 19-9;
